# Supplementary material for: Strategies of Modelling Incident Outcomes Using Cox Regression to Estimate the Population Attributable Risk
Source: Int J Environ Res Public Health. 2023 Jul 20;20(14):6417. doi: 10.3390/ijerph20146417 (PMC10379285; doi:10.3390/ijerph20146417)
Supplement: Supplementary file 1 [file ijerph-20-06417-s001.zip › Supplementary Table S1.pdf]

Supplementary Table S1. Pairwise Spearman correlation coefficients between age, sex, low physical activity, smoking, alcohol use and hypertension.

Variables	R_{Sp} (95% CI)	*Pvalue
AGE vs. SEX	-0.033 (-0.078, 0.011)	0.1425
**AGE vs. LPA**	**0.217 (0.174, 0.259)**	**<.0001**
AGE vs. SMK	0.012 (-0.033, 0.056)	0.6116
AGE vs. ALC	0.008 (-0.037, 0.052)	0.742
**AGE vs. HYT**	**0.251 (0.209, 0.292)**	**<.0001**
SEX vs. LPA	-0.067 (-0.111, -0.022)	0.0033
**SEX vs. SMK**	**-0.164 (-0.207, -0.120)**	**<.0001**
**SEX vs. ALC**	**-0.337 (-0.376, -0.296)**	**<.0001**
SEX vs. HYT	0.010 (-0.035, 0.054)	0.6728
LPA vs. SMK	0.027 (-0.018, 0.072)	0.2395
**LPA vs. ALC**	**0.136 (0.091, 0.179)**	**<.0001**
**LPA vs. HYT**	**0.110 (0.066, 0.154)**	**<.0001**
**SMK vs. ALC**	**0.439 (0.402, 0.474)**	**<.0001**
SMK vs. HYT	0.017 (-0.028, 0.062)	0.4585
**ALC vs. HYT**	**0.129 (0.085, 0.173)**	**<.0001**

*****Pvalue for H0: $R_{Sp} = 0$, **AGE**: Baseline age coded as continuous, **SEX**: Women coded as 1, **LPA**: Low physical activity Index, **SMK**: ever smoking, **ALC**: ever use of alcohol, **HYT**: hypertension
